# X-ray irradiation induced Disabled-2 gene promoter de-methylation enhances radiosensitivity of non-small-cell lung carcinoma cells

**DOI:** 10.1186/s13046-018-1000-3

**Published:** 2018-12-14

**Authors:** Shuang Ma, Wan-Lin Zhang, Bruce D. Leckey, Hong-Tao Xu, Lian-He Yang, Endi Wang

**Affiliations:** 1 Department of Neurology, Shengjing Hospital of China Medical University, Shenyang, 110022 Liaoning China; 2 Department of Pathology, the College of Basic Medical Sciences and First Affiliated Hospital of China Medical University, Shenyang, 110013 Liaoning China; 3 Department of Pathology, Duke University Medical Center, Durham, NC 27710 USA

**Keywords:** Disabled-2, Lung cancer, Wnt pathway, Radiotherapy, Radiosensitivity

## Abstract

**Background:**

Disabled-2 (Dab2) is known as a tumor suppressor as well as a Wnt pathway inhibitor. We previously reported that Dab2 was down-regulated due to gene promoter hypermethylation in lung cancer. Here, we aim to study if X-ray irradiation can induce de-methylation of the Dab2 gene and subsequently up-regulate its expression, and also to attempt to suppress the malignant biological behavior of and enhance the radiosensitivity in lung cancer cells with hypermethylation of the Dab2 gene.

**Methods:**

Immunostaining was performed to investigate the relationship between Dab2 expression and lung cancer clinicopathological characteristics. Bisulfite sequencing PCR (BSP) was used to evaluate the methylation status of lung cancer cells with or without X-ray treatment. Real-time PCR and western Blot were performed to investigate the expression of Dab2, Wnt pathway factors, DNMTs and methyl CpG binding protein 2 (MeCP2). Colony Formation, matrigel invasion and xenograft experiment were performed to evaluate the malignant biological behavior of lung cancer cells with irradiation.

**Results:**

The result of immunostaining of Dab2 in lung cancer tissues showed that decreased Dab2 expression was positively correlated with poor differentiation, lymph node metastasis, advanced TNM stage and poor prognosis. X-ray treatment significantly up-regulated Dab2 expression and inhibited Wnt factors in LK2 cells (with hypermethylation of the Dab2 gene promoter, *P* < 0.05), but not in SPC-A-1 cells (with hypomethylation of the Dab2 gene promoter); however, the effect could be reversed by Dab2 or Axin knockdown (*P* < 0.05). Decreased expression of DNMT1, DNMT3b and MeCP2 could be detected in both LK2 and SPC-A-1 cells compared to non-irradiated cells (*P* < 0.05). Both in vitro studies and in vivo xenograft tumor growth demonstrated that X-ray could significantly inhibit the proliferation and invasion of LK2 but not SPC-A-1 cells (*P* < 0.05).

**Conclusion:**

In general, X-ray-induced up-regulation of Dab2 and inhibition of the Wnt pathway may be mediated by de-methylation of a hypermethylated Dab2 gene promoter. X-ray treatment significantly inhibits proliferation and invasion of lung cancer cells with hypermethylation of the Dab2 gene promoter, but is less effective in lung cancer cells with hypomethylation of the Dab2 gene promoter. These results indicate that the methylation status of the Dab2 gene promoter might be a potential predictor of the radiosensitivity of lung cancer cells.

## Background

Abnormal Wnt/Wingless signaling pathway activation has been reported in many cancers and is supposed to promote proliferation, invasion, and metastasis [1, 2]. Wnt/β-catenin signaling is active during embryogenesis facilitating new organism formation. Normally, it is down-regulated in differentiated cells, however, the deregulated Wnt signaling is correlated with almost all stages of oncogenesis, from malignant transformation to metastatic dissemination and resistance to treatment [3, 4], it also induces radioresistance in several human cancers including head and neck, breast, nasopharyngeal, esophageal, glioblastoma, and colorectal cancers [5]. Abnormalities of Wnt components such as: mutation in β-catenin or APC gene, overexpression of Wnt ligands, loss of inhibition of regulatory could induce the activity and stimulates expression of downstream genes, such as: cyclin D1 and c-myc, matrix metalloproteinase-7 (MMP-7), connexin 42, CD44, claudin-1 and so on [6–9]. The overexpression of its downstream targets is correlated with the alteration of cell cycle progression, proliferation, invasiveness, Epithelial-mesenchymal transition (EMT), cancer stem cells (CSCs) and contribute to tumorigenesis [6–9].

Disabled-2 (Dab2) is a member of the Mammalian/Drosophila disabled gene family, which can stabilizes Axin and serves as an inhibitor of the Wnt pathway [10–14]. Lung cancer is now the leading cause of malignant tumor related deaths in China and the rest of the world [15, 16]. We previously reported that the Wnt signaling pathway is abnormally activated in lung cancer due to decreased expression of Dab2 and Axin, which inhibits the degradation of β-catenin resulting in its accumulation in the cytoplasm and nucleus. This accumulation of β-catenin then activates the target genes of the Wnt pathway, such as cyclin D1, MMP-7 and c-myc. We also have previously found that hypermethylation of the Dab2 gene promoter was correlated with reduced expression in lung cancer [17–19].

Non-small cell lung cancer (NSCLC) accounts for 85% of lung cancer cases. Radiotherapy is an important treatment for lung cancer patients, especially for patients who are not suitable for surgery. However, it is well known that lung cancers from different patients may display different degrees of radiosensitivity. Interestingly, X-ray irradiation has been shown to induce demethylation of the whole genome by inhibiting DNA methyltransferases (DNMTs) [20–25], which raise the possibility that X-ray irradiation might inhibit lung cancer cell proliferation and invasion via demethylation-mediated up-regulation of the Dab2 gene. In order to confirm our hypothesis, we assessed the methylation status of the Dab2 gene promoter and investigated its transcriptional expression after irradiation. In addition, we studied the effects of X-ray irradiation on expression of DNMTs, Wnt pathway factors, the methylation status of the Dab2 gene, as well as associated changes in cell proliferation, invasiveness, and tumor progression by in vitro and in vivo experiments.

## Methods

### Lung cancer tissue samples and immunohistochemistry

A total of 137 lung cancer tissue samples, including 63 squamous cell carcinoma and 74 adenocarcinoma cases, and the corresponding normal lung tissues were obtained randomly from patients who underwent surgery at the First Affiliated Hospital of China Medical University between 2009 and 2011. The follow-up study lasted for 60 months (4–60 months, average follow-up time 30.65 months). Patients included in the study ranged from 32 to 82 years of age (mean, 59 years), and were composed of 77 males and 60 females. These tumors showed different degrees of differentiation and were classified as well (*n* = 30), moderately (*n* = 67), or poorly (*n* = 40) differentiated. 65 cases exhibited lymph node metastasis. The tumor stage was classified according to the TNM classification system of the International Union Against Cancer as stage I (57 cases), stage II (52 cases), and stage III (28 cases). The study was conducted according to the regulations stipulated by the institutional review board at the China Medical University and complied with Declaration of Helsinki.

Sections were immunostained with polyclonal rabbit anti-Dab2 antibody (1400; Abcam Biotechnology Inc., Cambridge, MA), at 4 °C overnight. Antibodies were detected by the streptavidin-peroxidase method. The immunostained slides were reviewed by 2 pathologists who were blinded to the clinical data. Five fields of view per slide were randomly examined at 400× magnification. The positive rate of each case was obtained by calculating the percentage of positively stained cells on each slide. Percentages were scored as (1) 1 to 25%, (2) 26 to 50%, (3) 51 to 75%, or (4) 76 to 100%. The intensity of Dab2 staining was scored as 0 (negative), 1 (weak), 2 (moderate), and 3 (strong). The two scores were multiplied to give a final score from 0 to 12. The overall expression of Dab2 in these tumors was then defined as low expression (< 5) and high expression (≥5).

### Cell culture and X-ray treatment

Four cell lines of NSCLC, including LTEP-a-2 (LTE, adenocarcinoma), and NCI-H460 (H460, large cell carcinoma), obtained from the ATCC (Manassas, VA, USA); SPC-A-1 (SPC, adenocarcinoma) and LK2 (LK, squamous carcinoma) cell lines were obtained from the Cell Bank of the Chinese Academy (Shanghai, China). Plastic flasks with lung cancer cells were treated with X-ray irradiation using a linear accelerator (Primus, Siemens, Germany) with a dose of 2Gy and 4Gy. X-ray irradiation was delivered soon after the cell density reached 70–80%, and a non-irradiated lung cancer cell line was used as control. After irradiation, the cells were harvested at the appropriate time points and stored in a freezer (− 80 °C) before being processed for further analysis.

### Bisulfite sequencing PCR, real-time PCR and western blot

The genomic DNA from lung cancer cells either treated or untreated with X-ray irradiation was isolated by using a DNA extraction kit (TIANGEN BIOTECH BEIJING CO., LTD) according to the manufacturer’s instructions. DNA methylation kit was used to treat DNA samples (ZYMO RESEARCH Inc., USA). The methylation status of the region was examined by bisulfite sequencing PCR (BSP) using primers as follows: 5′-ATTTTTGGGGAGTTTTTAGTTG-3′ (forward) and 5′-ATGAGATTTTGTGTTTGGGGAA-3′ (reverse). PCR products were then separated by 3% agarose gel electrophoresis and were then cut from gels for purification using the E.Z.N.A Gel Extraction Kit (Omega Bio-tek, Doraville, GA). The purified products were subsequently cloned into pMD 19-T cloning vector (Takara), and at last, 6 clones were subjected to DNA sequencing (Wanleibio, Shenyang, China) after bacterial amplification. The AMF was defined as the value of mean methylation frequency the 23 CpG sites.

Real-time PCR (Fluorescent dye method, ABI, 7900HT) was performed to evaluate the transcripts of Dab2. The experiments were performed according to the manufactures’ instructions (SYBR® Premix Ex Taq™, Takara Bio) using PCR primers as follows: 5’-CTCGCTTCACGGAGGCAATAG-3′ (forward) and 5’-AGCAAACCTCAGTACCAGTGGACA-3′ (reverse). The experiment was repeated three times, and a mean value was obtained.

Antibodies against Dab2 (ab33441, 1:800, Abcam), Axin (C76H11, 1:500, cell signaling), β-catenin (562,505, 1:800, BD), cyclin D1 (H-295, 1:500, Santa), MMP-7 (sc-30,071, 1:500, Santa), c-myc (D3N8F, 1:800, cell signaling), and β-actin (14C10, 1:1000, cell signaling) were used in this study. The protein bands were visualized using ECL (Pierce, Rockford, IL, USA) and quantified using the DNR Bio-Imaging System. A mean value was calculated by three times repeated experiment.

### Colony formation and matrigel invasion

LK and SPC cell lines were used in Colony Formation assays. The process was performed as follows: 600 cells were grown in a 60 mm dish with culture medium. The cells were treated with 4Gy irradiation, 4Gy irradiation and Dab2 siRNA transfection, 4Gy irradiation and Axin siRNA transfection respectively, and untreated cells were used as a control group. After 24 h of incubation, the cells were then continuously cultured until visible colonies were formed (14 days). Only those containing ≥50 cells were counted. The rate of colony formation was indicated by the ratio of the number of clones over the number of seeded cells. The experiment was repeated three times, and a mean value was calculated. Matrigel cell invasion assay: LK and SPC cell lines were used and the groups were classified as mentioned in the Colony Formation assay. In each upper chamber, 5 × 10^5^ cells were grown in serum-free culture medium. The lower chambers were filled with medium containing 10% fetal calf serum. After incubation for 24 h, the cells that migrated through the pores were fixed with methanol for 30 min and stained with hematoxylin. For each filter, the number of cells was counted microscopically in 5 random fields under 200× magnification. A mean value was calculated by three times repeated experiment.

### Xenograft to nude mice

Four-week-old male BALB/c nude mice were obtained from the animal facility (Shanghai Slake Experimental Animal Co., Ltd.). The mice were handled in strict accordance with the recommendations in the Guide for the Care and Use of Laboratory Animals of the National Institutes of Health [26]. The protocol was approved by the Committee on the Ethics of Animal Experiments of the China Medical University and complied with Declaration of Helsinki. The mice were randomly divided into 4 groups (5 mice in each group, weight 17.34–22.58 g). Each mouse was inoculated subcutaneously in the right axilla with 5 × 10^6^ human lung cancer cells suspended in 0.2 ml sterile PBS. The large dimension (L) and short dimension (W) of the subcutaneous nodules were measured with vernier calipers every week. The tumor volume was calculated by the following formula: V=W^2^ × L× π/6, before plotting into the growth curve for each group. Five weeks after inoculation, the mice were sacrificed and the tumor nodules from each mouse were completely excised and measured. The rate of tumor growth inhibition (%) was calculated according to the formula: (mean tumor weight of control group-mean tumor weight of X-ray irradiation group)/mean tumor weight of control group× 100%.

### Statistical analysis

SPSS version 22 for Windows was utilized to analyze the data. Survival probability was analyzed by Kaplan-Meier analysis. The relationship between Dab2 expression and lung cancer patients’ clinical and pathological characteristic was analyzed by the Chi-Squared test. The Student’s t test and Mann–Whitney U test were used to evaluate statistical difference of other experimental data.

## Results

### Low Dab2 expression in lung cancer correlates with differentiation, TNM stage, lymph node metastasis and poor outcome

**Fig. 1:**
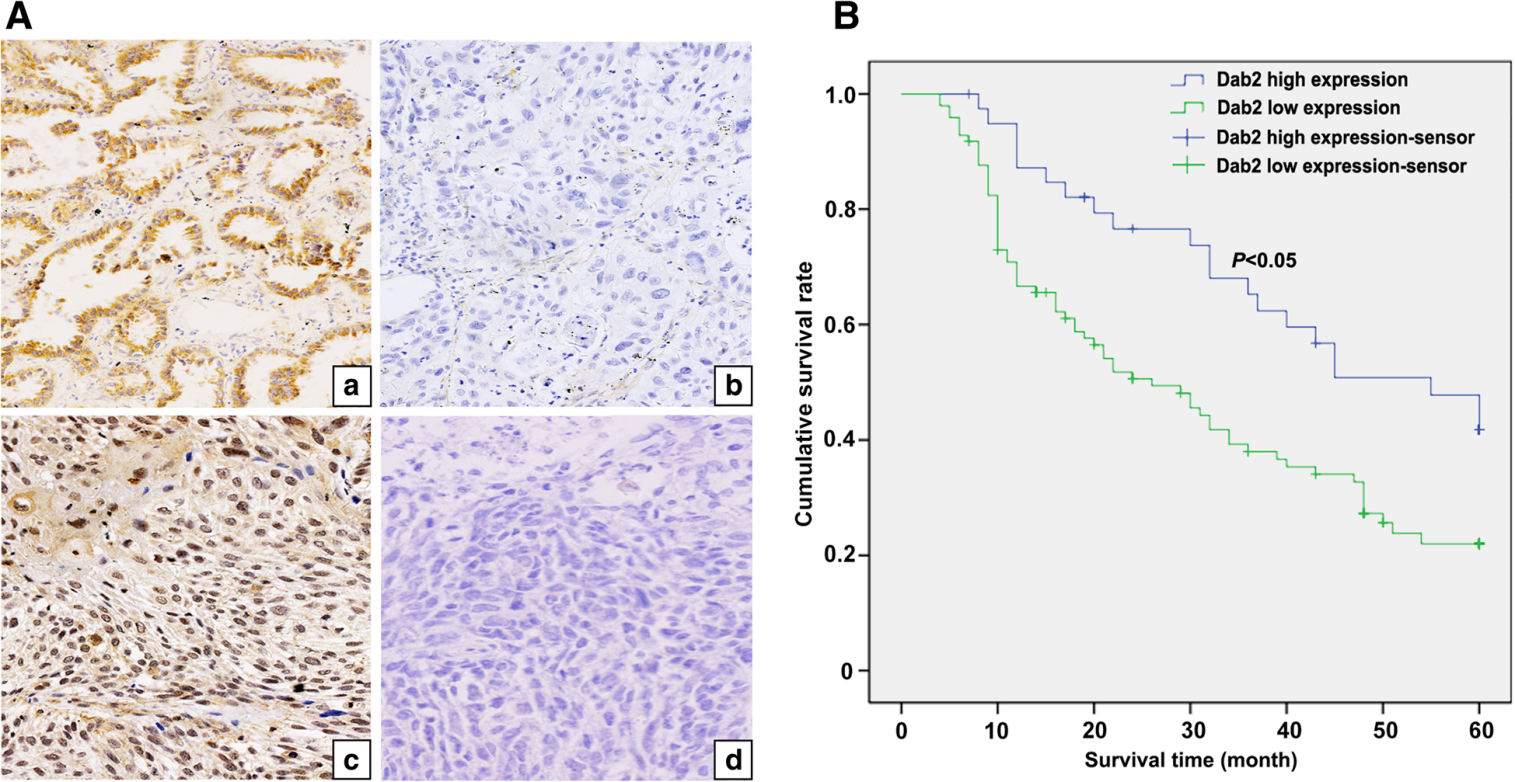
Correlation between Dab2 expression and lung cancer clinical characteristics as well as lung cancer patients’ outcome. **a** High cytoplasmic expression of Dab2 in well-differentiated adenocarcinoma cells (*a*, IHC, 400×); Negative expression of Dab2 in poorly-differentiated adenocarcinoma cells (*b*, IHC, 400×); Medium cytoplasmic and strong nuclear expression of Dab2 in well-differentiated squamous cell carcinoma cells (*c*, IHC, 400×); Negative expression of Dab2 in poorly-differentiated squamous cell carcinoma cells (*d*, IHC, 400×). **b** Kaplan-Meier analysis demonstrates that low Dab2 expression is positively correlated with poor outcome of lung cancer patients (*P* < 0.05)

Immunohistochemical staining for Dab2 revealed low expression in 97 cases and high expression in 40 cases. Low expression of Dab2 is not correlated with the patients’ age (*P* = 0.8692), gender (*P* = 0.5747) or histological classification (*P* = 0.5991), but has a close relationship with the differentiation (*P* < 0.0001), TNM stage (*P* = 0.006194) and lymph node metastasis (*P* = 0.00004, Table 1). The above results indicate that high Dab2 expression is negatively correlated with the progression of lung cancer (Fig. 1a). A survival analysis shows that the patients with high Dab2 expression have a better outcome than those patients with reduced Dab2 expression (*P* < 0.05, Fig. 1b).

**Table 1:** Correlations between Dab2 expression and lung cancer clinicopathological characteristics

	Quantity	Dab2 expression		χ^2^value	*P* value
		low	high		
Age					
<61	67	47	20	0.0271031	0.8692
≥ 61	70	50	20		
Gender					
Male	77	56	21	0.314948	0.5747
Female	60	41	19		
Histologic					
SCC	63	46	17	0.276302	0.5991
Adenocarcinoma	74	51	23		
Differentiation					
Well	30	8	22	36.7732	< 0.0001*
Moderate	67	54	13		
Poor	40	35	5		
TNM stage					
I	57	32	25	10.1682	0.006194*
II	52	42	10		
III	28	23	5		
Lymph node metastasis					
Yes	65	57	8	17.0663	0.00004*
No	72	40	32		

*presents *P* < 0.05

### Effect of X-ray irradiation on Dab2 mRNA and methylation level in lung cancer cells

**Fig. 2:**
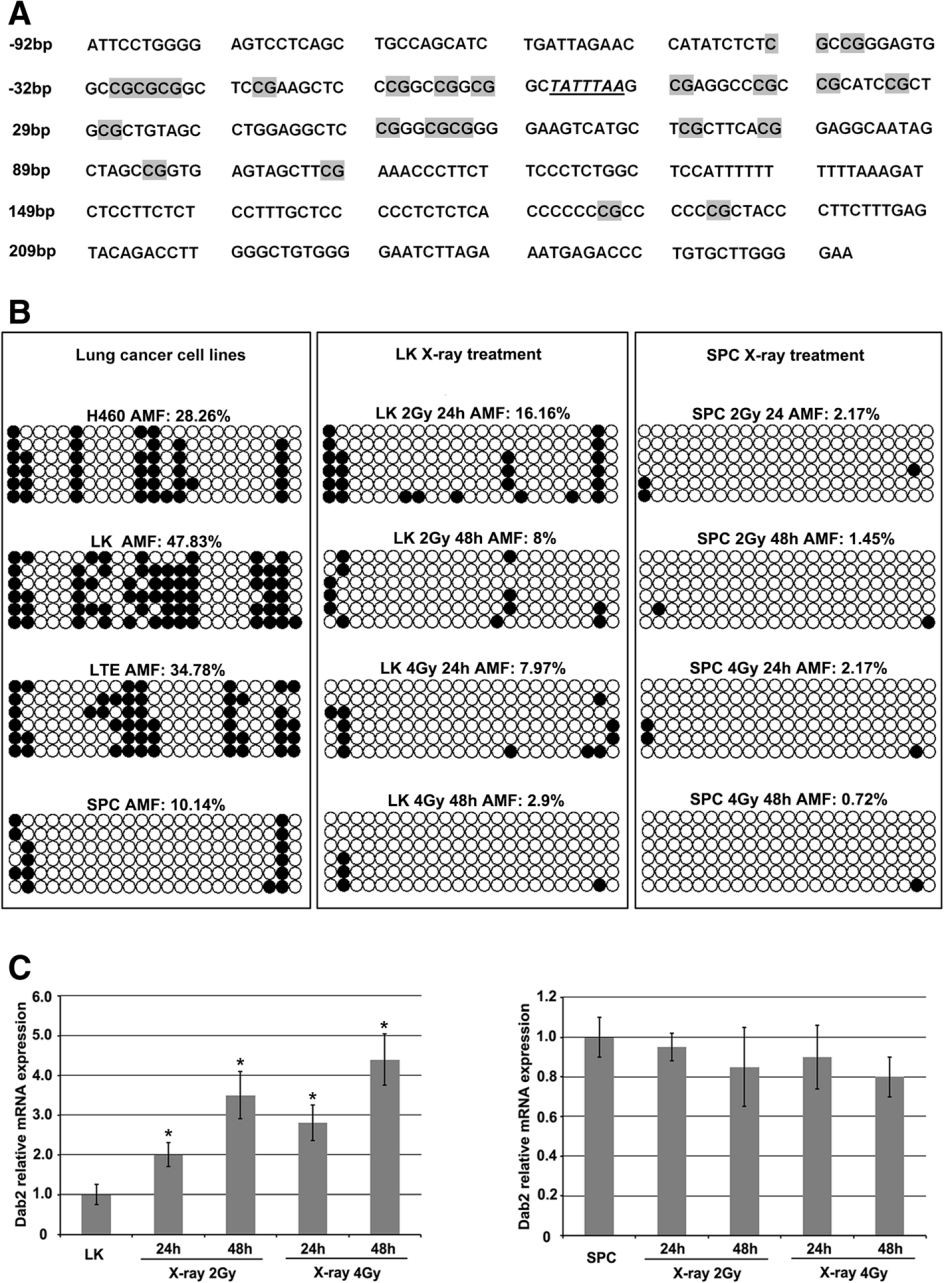
Effect of X-ray irradiation on Dab2 methylation and mRNA level in lung cancer cells with hypermethylation or hypomethylation of the Dab2 gene promoter. **a** Dab2 gene promoter contains a CpG island with 353 bp nucleotides and 23 CpG dinucleotides (highlighted with gray), transcription start site is highlighted with italics and underlined. **b** The AMF of non-irradiated lung cancer cells, X-ray irradiated LK and SPC cells are presented in the left, middle and right columns, respectively. LK and SPC cells were defined as hypermethylated or hypomethylated (47.83% VS 10.14%) according to the AMF. X-ray irradiation significantly induced de-methylation in LK and SPC cells compared with non-irradiated cells (black circle represents methylated CG dinucleotides, white circle represents unmethylated CG dinucleotides). **c** Real-time PCR showed that the Dab2 mRNA level was significantly up-regulated with irradiation of LK cells compared with non-irradiated LK cells (*P* < 0.05), but a slightly decreased level of Dab2 mRNA was observed in SPC cells with X-ray irradiation. * *P* < 0.05

A 353 bp sequence was assessed for average methylation frequency (AMF) in this study. This segment contains 23 CpG sites and includes a sequence of 92 bp upstream and 262 bp downstream of the transcription start site was presented (Fig. 2a) [12]. BSP shows the AMF of lung cancer cell lines as following: LK 47.83% (66/138), SPC 10.14% (14/138), H460 28.26% (39/138) and LTE 34.78% (48/138). Based on the AMF, LK and SPC were chosen as hypermethylated and hypomethylated cell lines for further studies, respectively (Fig. 2b, left column). BSP demonstrated that after 2Gy X-ray treatment (Fig. 2b, middle column), the AMF of the promoter regions of the Dab2 gene in LK cells was down-regulated significantly (*P* < 0.05), both at 24 h 16.16% (24/138) and 48 h 8% (11/138), compared to LK cells without X-ray treatment 47.83% (66/138). After 4Gy X-ray treatment, the AMF of the promoter regions of the Dab2 gene in LK cells was even more significantly down-regulated 7.97% (11/138) at 24 h and 2.9% (4/138) at 48 h, respectively (*P* < 0.05). We also identified down-regulation of methylation levels after irradiation in SPC cells, with 2.17% (3/138) at 24 h and 1.45% (2/138) at 48 h after 2Gy irradiation; and 2.17% (3/138) at 24 h and 0.72% (1/138) at 48 h after 4Gy irradiation (Fig. 2b, right column), compared to the non-irradiated SPC cells 10.14% (*P* < 0.05).

**Fig. 3:**
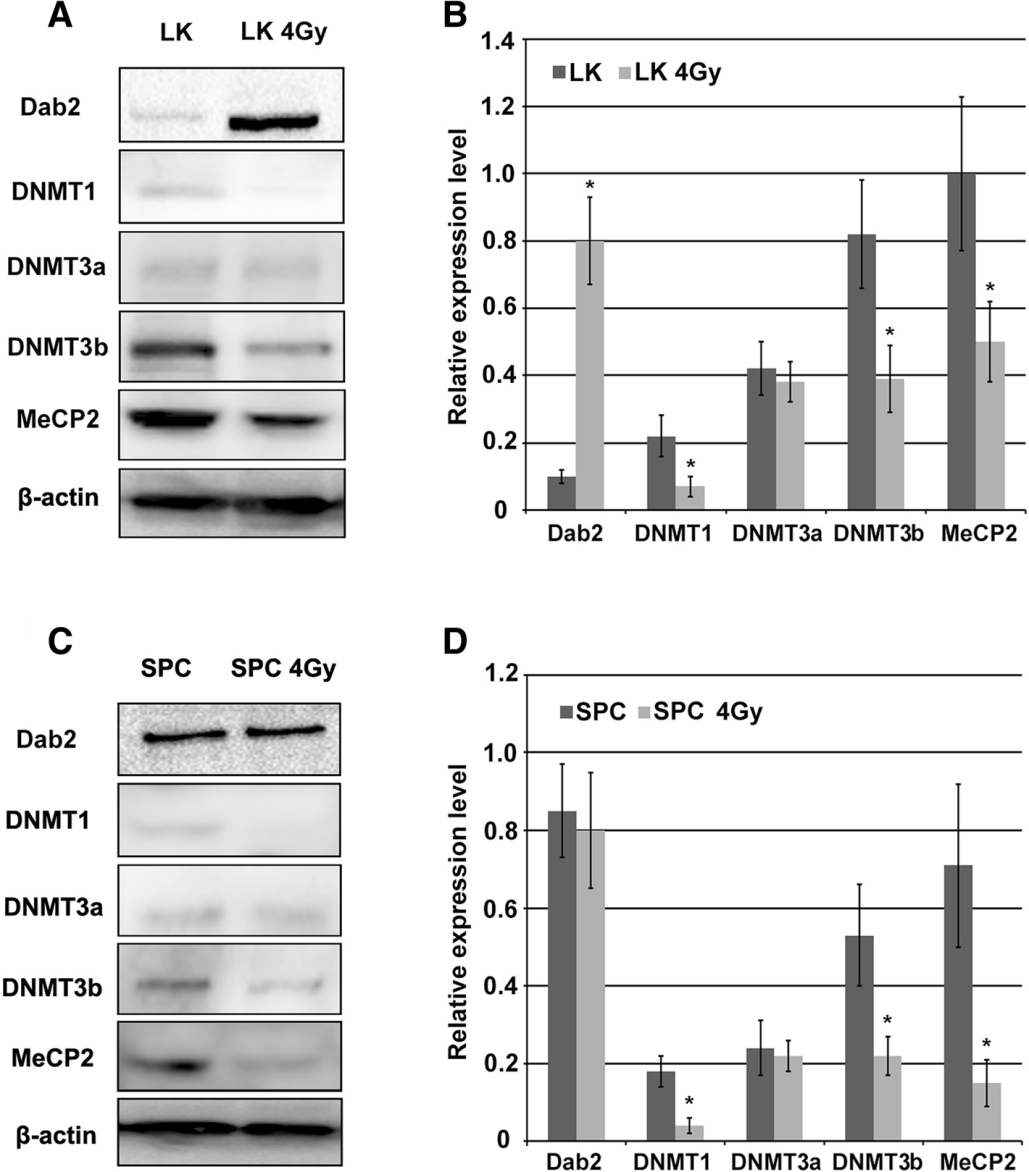
X-ray irradiation down-regulates DNMT1, DNMT3b and MeCP2 in lung cancer cells. **a** Western blot demonstrates the effect of X-ray irradiation on the expression of DNMTs and MeCP2. DNMT1, DNMT3b and MeCP2 were down-regulated significantly in irradiated LK cells compared with non-irradiated cells (*P* < 0.05), but not obvious change of DNMT3a expression was observed; **b** The histogram of A; **c** Decreased expression of DNMT1, DNMT3b and MeCP2 after X-ray irradiation in SPC cells (*P* < 0.05); **d** The histogram of C. * *P* < 0.05

Generally, Dab2 mRNA level was up-regulated in hypermethylated LK cells but not in hypomethylated SPC cells (Fig. 3c, *P* < 0.05). Real-time PCR results demonstrated that the Dab2 mRNA level was significantly up-regulated at 24 h and 48 h after 2Gy and 4Gy irradiation in LK cells (Fig. 2c, *P* < 0.05). The result is similar with the change of AMF in LK cells; however, no increased in Dab2 mRNA was observed after X-ray treatment in SPC cells, and there was a slight decreased of the Dab2 mRNA level (Fig. 2c). These results indicate that X-ray irradiation could up-regulate Dab2 mRNA level in cells with hypermethylation of the Dab2 gene promoter, but not in cells with hypomethylation of the Dab2 gene promoter.

### X-ray-induced DNMTs and MeCP2 down-regulation in lung cancer cells

We examined the protein levels of DNMTs (including DNMT1, 3a and 3b) at 24 h after 4Gy X-ray irradiation in LK and SPC cell lines. We noted that DNMT1 and DNMT3b were both significantly down-regulated in each cell line (Fig. 3, *P* < 0.05), but DNMT3a was not down-regulated. It is known that methyl CpG binding protein 2 (MeCP2) binds to DNA methyl groups and recruits histone deacetylase (HDAC), resulting in histone deacetylation, chromatin condensation and consequently, transcriptional inactivation of genes. Therefore, we examined the expression of MeCP2 and demonstrated a decrease in MeCP2 protein in LK and SPC cells, (Fig. 3a and b, *P* < 0.05). These results indicate that X-ray irradiation may down-regulate DNMT1, DNMT3b and MeCP2, and thus lead to transcriptional activation of the Dab2 gene in hypermethylated lung cancer cells.

### X-ray-induced increased Dab2 expression inhibits the Wnt pathway

**Fig. 4:**
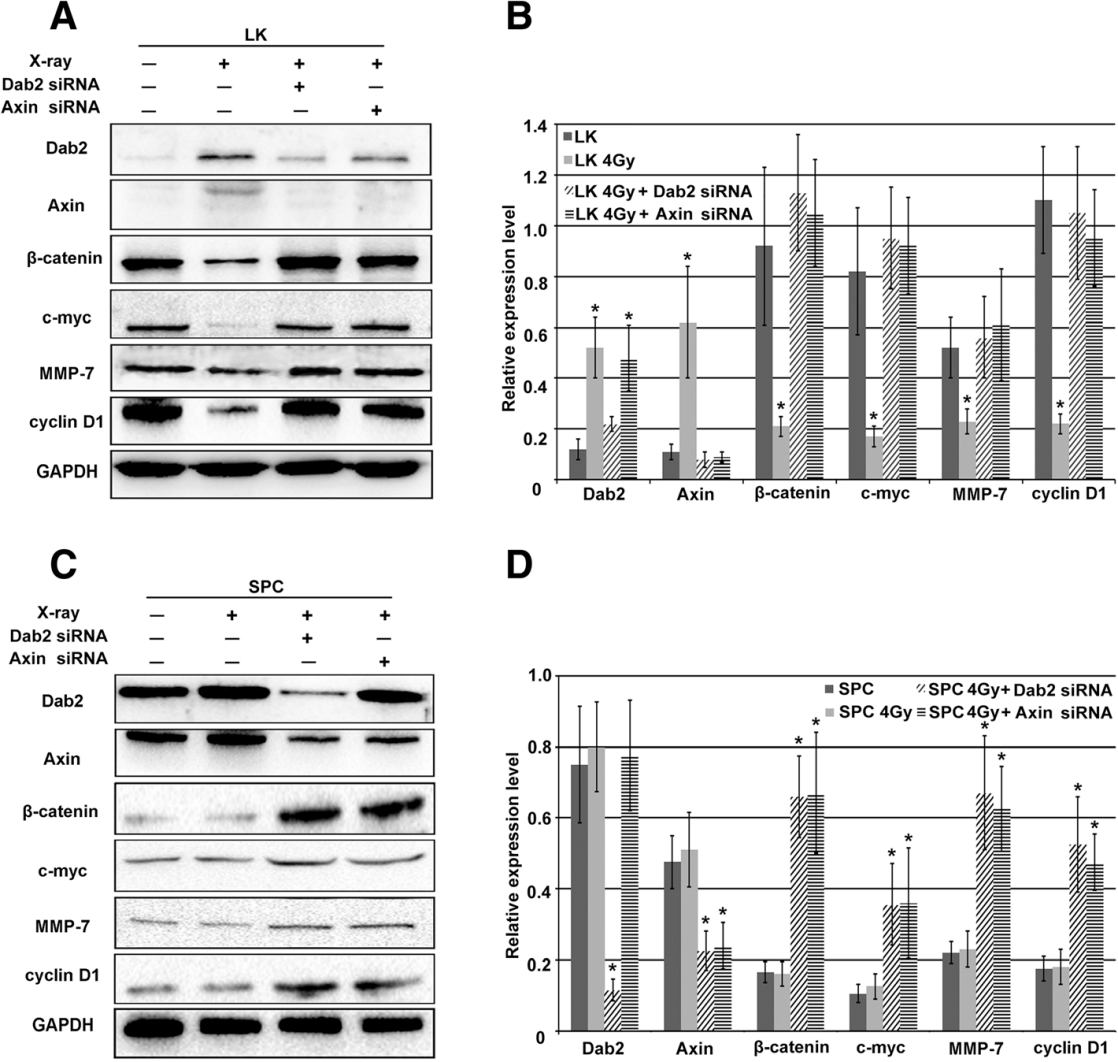
The effect of X-ray irradiation on Wnt pathway factors. **a** Western blot demonstrated increased Dab2 and Axin expression in LK cells with X-ray irradiation compared with non-irradiated cells (*P* < 0.05). Subsequent decreased expression of β-catenin and downstream Wnt pathway factors including cyclin D1, MMP-7 and c-myc were also detected (*P* < 0.05). The combination of X-ray irradiation and Dab2 siRNA transfection significantly reversed the up-regulation of Dab2 and Axin, as well as the decreased expression of β-catenin and downstream factors of the Wnt pathway (*P* < 0.05). The combination of X-ray irradiation and Axin siRNA did not influence Dab2 expression but significantly reversed the up-regulation of Axin, as well as the down-regulation of β-catenin and downstream factors of the Wnt pathway (*P* < 0.05); **b** The histogram of A; **c** X-ray irradiation did not influence the expression of Dab2 or Wnt pathway factor expression levels in irradiated SPC cells compared with non-irradiated cells, however, combination of X-ray irradiation and Dab2 siRNA significantly down-regulated Dab2 and Axin expression, and the expression of β-catenin, cyclin D1, MMP-7 and c-myc were up-regulated (*P* < 0.05). The combination of X-ray irradiation and Axin siRNA did not affect Dab2 expression but significantly down-regulated the expression of Axin, and subsequent up-regulation of β-catenin and downstream factors of the Wnt pathway was observed (*P* < 0.05); **d** The histogram of C. * *P* < 0.05

It has been reported that Dab2 can stabilize Axin and attenuate the classic Wnt/β-catenin signaling pathway. Axin is a negative regulator of the classic Wnt pathway, while β-catenin is the key positive regulator of the Wnt pathway. Cyclin D1 and MMP-7 are important downstream factors of the Wnt pathway which correlate with cell proliferation and invasion [10–14, 17–19, 27]. We noted that Dab2 was up-regulated in LK cells after X-ray treatment, but the expression of β-catenin, cyclin D1 and MMP-7 were down-regulated significantly (Fig. 4a and b, *P* < 0.05); Dab2 siRNA or Axin siRNA transfection were performed to identify whether X-ray induced Dab2 expression could inhibit the classic Wnt pathway. Combination of X-ray irradiation with Dab2 siRNA demonstrated only slight increased Dab2 expression while there was a slight decrease in Axin expression compared to X-ray irradiation alone. β-catenin, cyclin D1 and MMP-7 all demonstrated increased expression with combination of X-ray irradiation and Dab2 siRNA (Fig. 4a and b, *P* < 0.05). These results indicate that X-ray irradiation leads to the inhibition of the Wnt pathway via increased Dab2 expression in LK cells. Interestingly, a similar effect was observed with the combination of X-ray irradiation and Axin siRNA (Fig. 4a and b).

For SPC cells, X-ray irradiation showed almost no effect on the expression of Dab2 and other factors. Combination of X-ray irradiation and Dab2 siRNA lead to significantly decreased levels of Dab2 and Axin, while the expression of β-catenin, cyclin D1 and MMP-7 all significantly increased (Fig. 4c and d, *P* < 0.05). Combination of X-ray irradiation and Axin siRNA has almost no effect on Dab2 levels but lead to significantly decreased levels of Axin, while again the expression of β-catenin, cyclin D1 and MMP-7 all significantly increased (Fig. 4c and d, *P* < 0.05). These results indicate that X-ray irradiation in SPC cell lines does not induce up-regulation of Dab2, and that Dab2 knockdown leads to increased Wnt pathway signaling via the inhibition of Axin. Overall, the results in LK and SPC cells suggest that X-ray irradiation inhibits the classic Wnt pathway via increased expression of Dab2, which is probably the result of de-methylation of the Dab2 gene promoter due to the irradiation.

### X-ray irradiation significantly inhibited the proliferation and invasion in lung cancer cells with hypermethylation of the Dab2 gene promoter

**Fig. 5:**
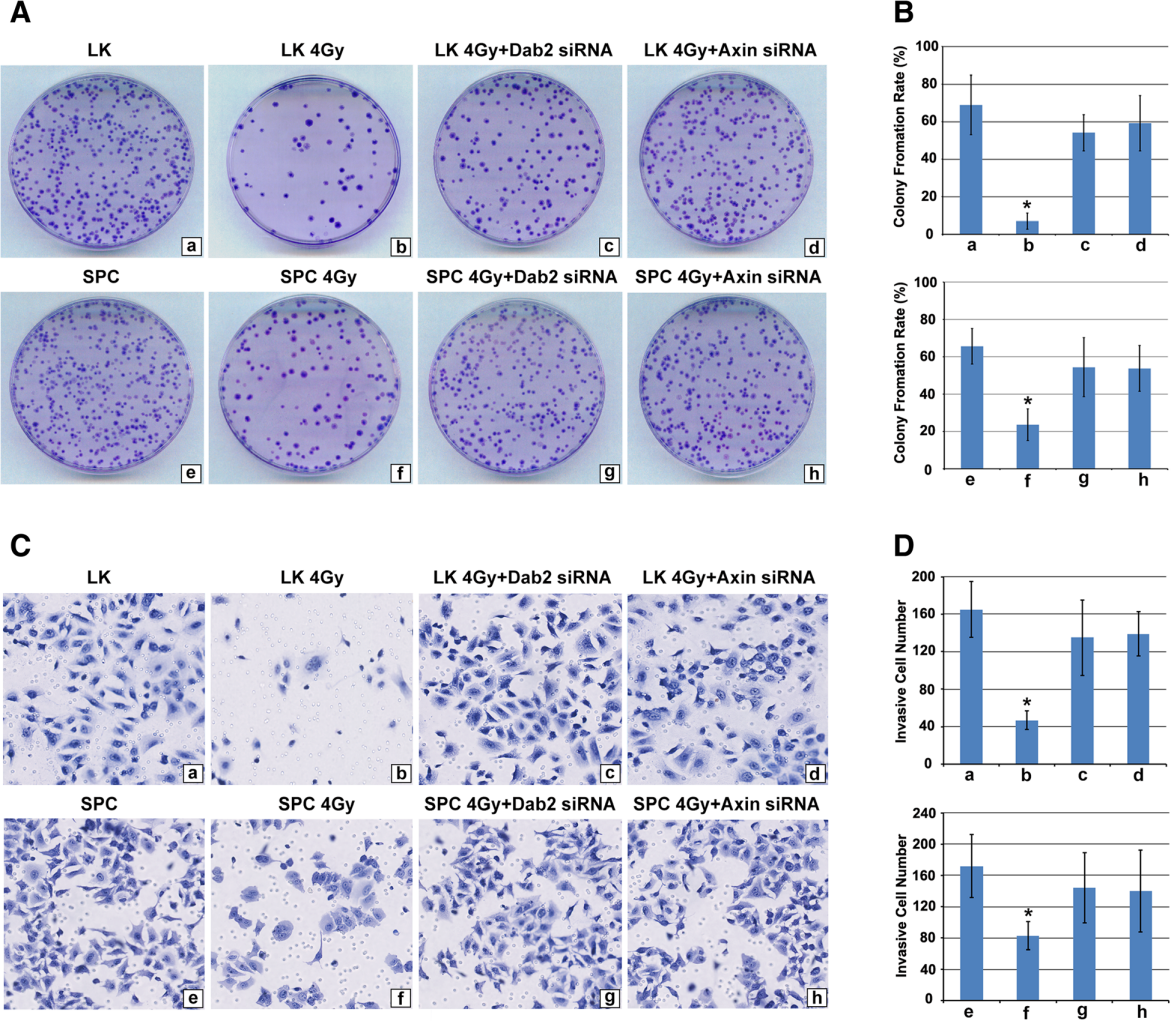
The effect of X-ray Irradiation on colony formation and invasion of lung cancer cells with hypermethylation or hypomethylation of the Dab2 gene promoter. **a** X-ray irradiation induced significant down-regulation of colony formation in irradiated LK cells compared with non-irradiated LK cells (*a* and *b*, 65.89% ± 15.83% VS 7% ± 4.2%, *P* < 0.05), however, the combination of X-ray irradiation and Dab2 knockdown significantly reversed this down-regulation compared with X-ray treatment alone (*c*, 54.11 ± 9.6%, *P* < 0.05). A similar effect was observed with combination of X-ray irradiation and Axin knockdown (*d*, 59.22 ± 14.73%, *P* < 0.05); However, X-ray irradiation alone was less effective in SPC cells compared with non-irradiated cells (*e* and *f*, 65.67 ± 9.54% VS 23.67 ± 8.51%, *P* < 0.05). Combination of X-ray irradiation and Dab2 siRNA (*g*, 54.44 ± 15.74%) or Axin siRNA (*h*, 53.78 ± 12.19%) also effectively reversed the down-regulation of clonal rate in SPC cells (*P* < 0.05); **b** The histogram of A; **c** Transwell cell invasion experiment demonstrated that the number of invasive LK cells decreased significantly after X-ray irradiation (*b*) compared to the non-irradiated group (*a*) (47 ± 10 VS 165 ± 30, *P* < 0.05). Combination of X-ray irradiation and Dab2 siRNA (*c*, 135 ± 40) or Axin siRNA (*d*, 139 ± 24) reversed the decreased invasive ability of LK cells (*P* < 0.05). The number of invasive SPC cells with irradiation (*b*, 83 ± 18) was less than that in non-irradiated SPC cells (*a*, 172 ± 40). X-ray irradiation induced decreased invasive ability of SPC cells was reversed by the transfection of Dab2 siRNA (*c*, 144 ± 45) and Axin siRNA (*d*, 140 ± 52); **d** The histogram of C. * *P* < 0.05

LK and SPC cells were treated with 4Gy X-ray irradiation, 4Gy X-ray irradiation and Dab2 knockdown, 4Gy X-ray irradiation and Axin knockdown, respectively, with non-irradiated cells serving as a control. For LK cells, colony formation rate was 68.89% ± 15.83% for the control group (Fig. 5a, *a*) and 7.2% ± 4.2% for X-ray irradiation alone (Fig. 5a, *b*), but combination of X-ray irradiation and Dab2 siRNA lead to reversal of the deceased colony formation rate observed with X-ray irradiation alone (54.11% ± 9.6%, Fig. 5a, *c*, *P* < 0.05). A similar effect was observed for the combination of X-ray irradiation and Axin siRNA (59.22% ± 14.73%, Fig. 5a, *d*, *P* < 0.05). In contrast, X-ray irradiation was less effective in SPC cells with the efficacy of colony formation being 65.67% ± 9.54% for the control group and 23.67% ± 8.51% for X-ray irradiation alone (Fig. 5a, *e* and *f*), respectively. Interestingly, more remarkable reduction of colony formation could be seen in LK cells than that in SPC cells after irradiation (*P* < 0.05). Combination of X-ray irradiation and Dab2 siRNA in SPC cells resulted in reversal of the deceased colony formation rate observed with X-ray irradiation alone (54.44% ± 15.74%, Fig. 5a, *g*), as well as the combination use of X-ray irradiation and Axin siRNA (53.78% ± 12.19%, Fig. 5a, *h*). These results suggest that X-ray irradiation significantly reduced the proliferation of lung cancer cells with hypermethylation of the Dab2 gene promoter, but is less effective in lung cancer cells with hypomethylation of the Dab2 gene promoter. Dab2 or Axin knockdown significantly decreased the ability of X-ray irradiation to reduce the proliferative activity of lung cancer cells.

We then assessed what effect X-ray irradiation has using the transwell cell invasive assay performed with LK and SPC cell lines and the same group classifications. After X-ray irradiation alone, the number of invasive cells of the LK cell line was significantly decreased compared to the control group (Fig. 5c *a* and *b*; 165 ± 30 VS 47 ± 10, *P* < 0.05), however, combination of X-ray irradiation and Dab2 siRNA or Axin siRNA reversed the effect (Fig. 5c *c* and *d*, 135 ± 40 and 139 ± 24, respectively). For the SPC cell line, X-ray irradiation alone also demonstrated a significant decrease in the number of invasive cells compared to the control group (Fig. 5c *e* and *f*, 172 ± 40 VS 83 ± 18, *P* < 0.05), but the effect was less than that of the LK cells (Fig. 5c *b* and *f*, and Fig. 5d *b* and *f*, *P* < 0.05). Combination of X-ray irradiation and Dab2 siRNA or Axin siRNA also reversed the effect in SPC cells (Fig. 5c, *g* and *h*, 144 ± 45 and 140 ± 52, *P* < 0.05). These results indicate that X-ray irradiation leads to decreased invasiveness in lung cancer cells with hypermethylation of the Dab2 gene promoter.

### X-ray irradiation inhibits xenograft tumor growth in lung cancer cells with hypermethylation of the Dab2 gene promoter

**Fig. 6:**
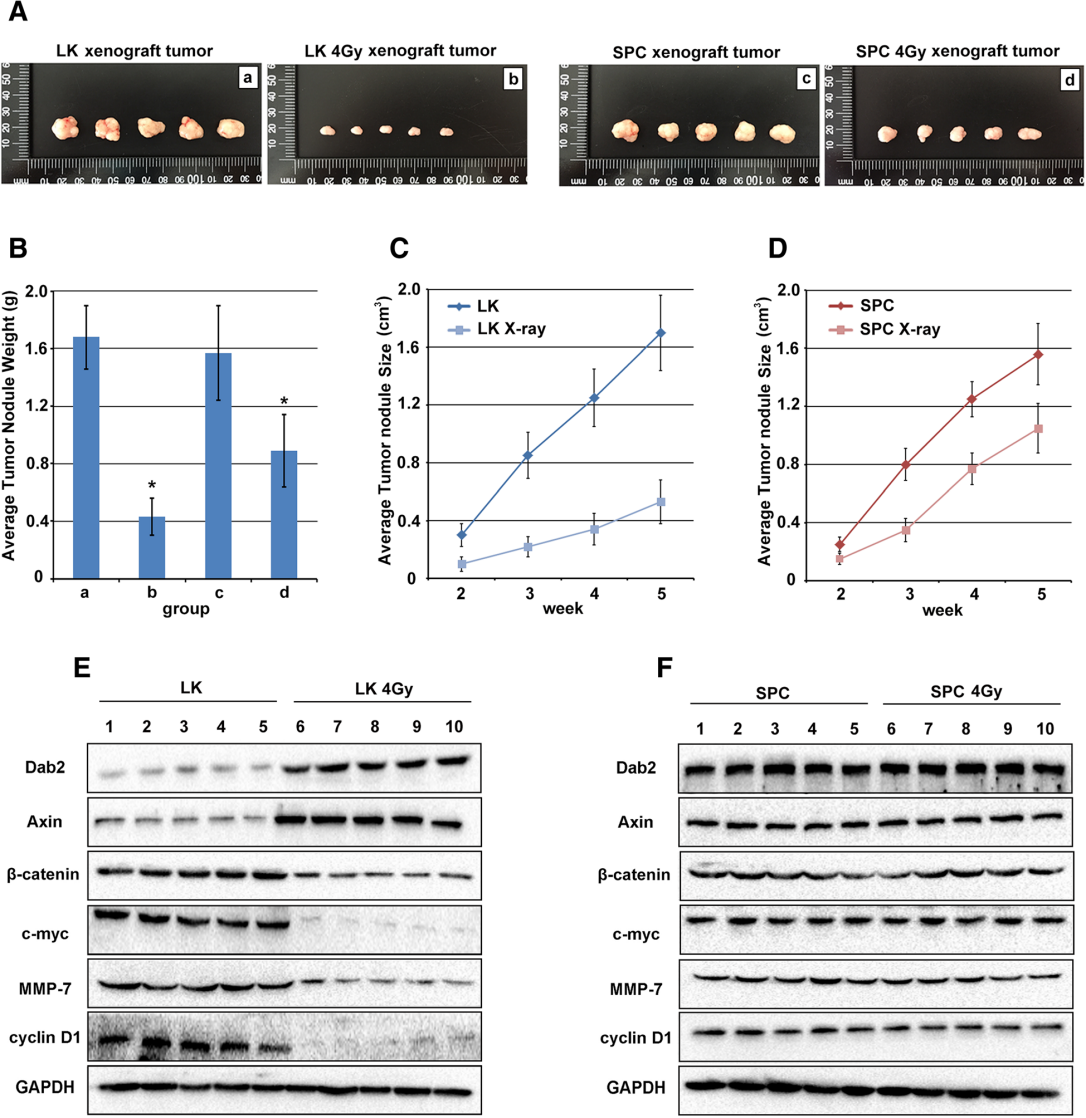
The effect of X-ray irradiation on xenograft tumor growth in LK and SPC cells. **a** Photograph of mice and excisional xenograft tumors inoculated with LK cells (*a*), 4Gy irradiation treated LK cells (*b*), SPC cells (*c*) and 4Gy irradiation treated SPC cells (*d*) for 5 weeks. More significant inhibition was observed in LK irradiated cells rather than SPC irradiated cells (*P* < 0.05). **b** Histogram representation of average tumor weight of each group. X-ray irradiation was more effective in LK cells rather than SPC cells (*P* < 0.05). **c** and **d**. Average tumor volumes of LK and SPC cells after irradiation, respectively. The results showed more significant inhibition in LK cells at 2, 3, 4 and 5 week than SPC cells (*P* < 0.05). **e** Dab2 and Axin were up-regulated in LK xenograft tumors with X-ray treatment comparing with un-treated group (1–5 VS 6–10 *P* < 0.05), and decreased expression of β-catenin, c-myc, MMP-7 and cyclinD1 were also identified in irradiation group but not in un-treated group (1–5 VS 6–10 *P* < 0.05). **f** No obvious difference of Dab2 and other factors of Wnt pathway were identified between SPC xenograft tumors with X-ray treatment and un-treated group (1–5 VS 6–10). * *P* < 0.05

LK and SPC cells, after 4Gy X-ray irradiation, were inoculated into nude mice in the right axilla, respectively. Non-irradiated cells served as control group. After 2 weeks, tumor nodule formation was observed and their volumes were measured weekly thereafter. The xenograft tumors were completely excised at 5 weeks (Fig. 6a). The results showed a remarkable reduction of the average tumor volume after comparing non-irradiated LK cells with the 4Gy treatment group (Fig. 6a, *a* and *b*, 1.7 ± 0.26 cm^3^ VS 0.53 ± 0.15 cm^3^, *P* < 0.05), while in SPC cells, X-ray irradiation was less effective after comparing non-irradiated SPC cells with the 4Gy treatment group (Fig. 6a *c* and *d*, 1.56 ± 0.21 cm^3^ VS 1.05 ± 0.17 cm^3^, *P* < 0.05). The average weight of the tumors were markedly reduced in LK cells receiving X-ray irradiation, from 1.68 ± 0.41 g to 0.43 ± 0.07 g (Fig. 6b, *P* < 0.05); however, irradiation was less effective in weight reduction in the SPC xenograft tumors (from 1.57 ± 0.33 g to 0.89 ± 0.25 g, *P* < 0.05). Comparison of the reduction rate of tumor weight in LK and SPC cells with X-ray irradiation (74.4% VS 43.31%, Fig. 6b, *P* < 0.05), demonstrates that LK cells with hypermethylation of the Dab2 gene promoter are more sensitive to X-ray treatment than SPC cells with hypomethylation of the Dab2 gene promoter. The average tumor size of LK and LK with 4Gy irradiation treatment at 2, 3, 4 and 5 weeks showed that the growth of the LK cell line was significantly reduced (Fig. 6c, *P* < 0.05), and although X-ray treatment reduced the growth of SPC cells, the effect was less significant (Fig. 6d, *P* < 0.05). Western blot was performed to evaluate the expression of Dab2, Axin and other Wnt factors in LK and SPC xenograft tumors with or without X-ray treatment. The result showed that average Dab2 and Axin expression were significantly up-regulated in irradiated LK xenograft tumors comparing with un-treated LK xenograft tumors (Fig. 6e, *P* < 0.05), and the expression of β-catenin, c-myc, MMP-7 and cyclinD1 were down-regulated in LK xenograft tumors with irradiation (Fig. 6e, *P* < 0.05), but no significant change was identified in SPC xenograft tumors with or without irradiation (Fig. 6f). Overall, X-ray irradiation demonstrated suppression of tumor growth in both cell lines, although the extent of suppression in LK cells was much more prominent than in SPC cells.

## Discussion

Lung cancer is common worldwide, and has replaced liver cancer as the number one cause of death among people with malignancy in China since 2008, with a 464.84% increased mortality rate during the past 3 decades [16]. Several studies indicate that X-ray irradiation leads to deceased methylation in normal mammalian tissues [28–31], however, little is known about the change of methylation status in lung cancer cells with X-ray irradiation treatment, as well as the correlation between the methylation status and radiosensitivity of lung cancer, especially of specific gene promoters. Interestingly, our results demonstrate that X-ray irradiation could significantly induce de-methylation of the Dab2 gene promoter.

It is well known that DNMTs are responsible for the methylated status of genes. DNMT1, DNMT3a and DNMT3b are recognized as three types of DNMTs in mammals [24, 28–31]. Raiche, J. et al. demonstrated that radiation-induced DNA methylation changes correlated with radiation-induced alterations in expression of DNMTs in mice, and radiation-induced expression of DNMTs presented tissue-specificity, with DNMT3a and DNMT3b being the most important in radiation-induced DNA methylation alterations [24]. Ilnytskyy, Y. et al. reported that global DNA methylation was paralleled by a decrease in the level of MeCP2 after X-ray irradiation in mice spleen [31]. We also evaluated the expression of MeCP2 with or without X-ray irradiation, and the result showed that MeCP2 was down-regulated significantly both in LK and SPC cells. Real-time PCR showed that X-ray irradiation up-regulated Dab2 mRNA in lung cancer cells with hypermethylation of the Dab2 gene promoter (LK) but not in lung cancer cells with hypomethylation of the Dab2 gene promoter (SPC). In general, we hypothesize that down-regulation of DNMT1, DNMT3b and MeCP2 closely correlates with de-methylation of the Dab2 gene promoter, as well as the up-regulation of Dab2 expression, but further study is still needed to clarify the exact mechanisms, because the effect of the changes of DNMTs and MeCP2 are not solely related to the Dab2 gene.

Our study indicates that hypermethylated Dab2 gene might lead to the active of Wnt pathway. We also found that X-ray irradiation up-regulated Dab2 expression in lung cancer cells with hypermethylated Dab2 gene, which lead to increased Axin expression and subsequently decreased expression of β-catenin, as well as Wnt pathway factors including: cyclin D1, MMP-7 and c-myc in LK cells. These results indicate that X-ray irradiation up-regulates Dab2 expression and inhibits the Wnt pathway in lung cancer cells with hypermethylation Dab2 gene promoter.

The in-vitro studies showed that X-ray irradiation significantly inhibited the proliferative and invasive ability of lung cancer cells with hypermethylation of the Dab2 gene promoter, but this effect could be reversed by the knockdown of Dab2 or Axin, which indicates that this process is via the classic Wnt pathway. In-vivo studies demonstrated that xenograft tumor growth was suppressed more significantly in LK cells with hypermethylation of the Dab2 gene promoter rather than SPC cells with hypomethylation of the Dab2 gene promoter, which indicates that radiosensitivity may be enhanced by X-ray induced de-methylation of the Dab2 gene promoter. Herein, we propose that different methylation states of the Dab2 gene promoter correlates with raidosensitivity of lung cancer cells, and that hypermethylation of the Dab2 gene promoter may potentially serve as a molecular pathologic marker for radiotherapy treatment. However, further investigation with more lung cancer cell lines should be used to confirm this hypothesis.

## Conclusion

In summary, X-ray treatment could down-regulate DNMTs and MeCP2, then induce de-methylation of hypermethylated Dab2 gene promoter and up-regulation of Dab2 expression in lung cancer cells, which is positively correlated with the inhibition of classic Wnt pathway. In vitro/vivo experiments demonstrate that the proliferative and invasive ability were significantly suppressed in lung cancer cells with hypermethylated Dab2 gene promoter with irradiation, but less effective in lung cancer cells with hypomethylated Dab2 gene promoter. These results suggest that the methylation status of Dab2 gene promoter might be a potential predictor of the radiosensitivity of lung cancer cells.

## Abbreviations

AMF: Average methylation frequency
BSP: Bisulfite sequencing PCR
CSCs: Cancer stem cells
Dab2: Disabled-2
DNMTs: DNA methyltransferases
EMT: Epithelial-mesenchymal transition
HDAC: Histone deacetylase histone deacetylase
MeCP2: Methyl CpG binding protein 2
MMP-7: Matrix metalloproteinase-7
NSCLC: Non-small cell lung cancer
